# Simple, Visual, Point-of-Care SARS-CoV-2 Detection Incorporating Recombinase Polymerase Amplification and Target DNA–Protein Crosslinking Enhanced Chemiluminescence

**DOI:** 10.3390/bios14030135

**Published:** 2024-03-06

**Authors:** Hui Chen, Zhiyuan Zhuang, Naihan Xu, Ying Feng, Kaixin Fang, Chunyan Tan, Ying Tan

**Affiliations:** 1 State Key Laboratory of Chemical Oncogenomics, Institute of Biomedical and Health Engineering, Shenzhen International Graduate School, Tsinghua University, Shenzhen 518055, China; hui-chen21@mails.tsinghua.edu.cn (H.C.); zzy_joseph@163.com (Z.Z.); fengy22@mails.tsinghua.edu.cn (Y.F.); fkx21@mails.tsinghua.edu.cn (K.F.); tancy@sz.tsinghua.edu.cn (C.T.); 2 Department of Chemistry, Tsinghua University, Beijing 100084, China; 3 School of Food and Drug, Shenzhen Polytechnic University, Shenzhen 518055, China

**Keywords:** SARS-CoV-2, DNA–protein crosslinking, RPA, chemiluminescence, visual detection

## Abstract

The ongoing COVID-19 pandemic, driven by persistent SARS-CoV-2 transmission, threatens human health worldwide, underscoring the urgent need for an efficient, low-cost, rapid SARS-CoV-2 detection method. Herein, we developed a point-of-care SARS-CoV-2 detection method incorporating recombinase polymerase amplification (RPA) and DNA–protein crosslinking chemiluminescence (DPCL) (RPADPCL). RPADPCL involves the crosslinking of biotinylated double-stranded RPA DNA products with horseradish peroxidase (HRP)-labeled streptavidin (SA-HRP). Modified products are captured using SA-labeled magnetic beads, and then analyzed using a chemiluminescence detector and smartphone after the addition of a chemiluminescent substrate. Under optimal conditions, the RPADPCL limit of detection (LOD) was observed to be 6 copies (within the linear detection range of 1–300 copies) for a plasmid containing the SARS-CoV-2 N gene and 15 copies (within the linear range of 10–500 copies) for in vitro transcribed (IVT) SARS-CoV-2 RNA. The proposed method is convenient, specific, visually intuitive, easy to use, and does not require external excitation. The effective RPADPCL detection of SARS-CoV-2 in complex matrix systems was verified by testing simulated clinical samples containing 10% human saliva or a virus transfer medium (VTM) spiked with a plasmid containing a SARS-CoV-2 N gene sequence or SARS-CoV-2 IVT RNA. Consequently, this method has great potential for detecting targets in clinical samples.

## 1. Introduction

The COVID-19 pandemic, caused by severe acute respiratory syndrome coronavirus type 2 (SARS-CoV-2), has resulted in over 760 million confirmed cases and more than 6.8 million deaths worldwide [1,2,3]. The SARS-CoV-2 genome contains six open reading frames (ORFs) encoding replicase (ORF1a/ORF1b), spike (S), envelope (E), membrane (M), and nucleocapsid (N) proteins [4,5]. Among these components, the S, M, E, and N proteins are key structural proteins that constitute the outer shell of the SARS-CoV-2 virion [6]. During the early stages of human SARS-CoV-2 infection, typical symptoms include fever and dry cough, which are often accompanied by pulmonary inflammation [7]. Importantly, older individuals (>60 years of age) with COVID-19 have a statistically significant increased risk of developing additional pathological conditions, some of which are life-threatening [8]. Consequently, SARS-CoV-2 infection represents a substantial threat to human health and life, underscoring the urgent need for accurate and sensitive SARS-CoV-2 detection methods to address this challenge [9].

Numerous SARS-CoV-2 detection methods that are currently under development for combating the COVID-19 pandemic incorporate viral isolation, molecular biological detection, and immunoassay techniques [10,11]. Among these approaches, reverse transcription-quantitative PCR (RT-qPCR) is the most widely used, due to its exceptional sensitivity and specificity [12]. However, RT-qPCR procedures typically involve intricate primer design steps, as well as requiring expensive equipment and sophisticated technological skills that render them time-consuming, costly, and impractical for on-site clinical SARS-CoV-2 detection [13,14,15]. In contrast, immunological detection platforms, particularly those based on lateral flow assays (LFAs), are commonly employed for SARS-CoV-2 detection, due to their speed, affordability, and ease of use [16,17,18]. Nonetheless, LFAs usually lack sufficient sensitivity and can produce false negative results. The exact viral load in infected patients ranges between 641 and 1.43 × 10^11^ copies/mL, with a median of 7.99 × 10^4^ in throat samples and 7.52 × 10^5^ in sputum samples, respectively [19]. In recent years, there has been a growing emphasis on smartphone-based SARS-CoV-2 gene detection, primarily driven by the convenience and ease of use offered by this approach [20]. Sensitive point-of-care strategies for SARS-CoV-2 detection are attracting increasing attention for use in clinical applications.

Recombinase polymerase amplification (RPA), a recently developed nucleic acid amplification method, has garnered significant attention, due to its exceptional sensitivity, rapid processing time, and simple implementation [21]. These attributes render RPA particularly well-suited for virus detection applications [22]. As a result, real-time RPA detection procedures [23,24], RPA/CRISPR/Cas systems [25,26], and RPA/LFA methods [27] have been incorporated in widely used clinical SARS-CoV-2 detection assays. However, real-time RPA and RPA/CRISPR/Cas methods require reporter systems with complex chemical structures, expensive reagents, and additional instrumentation for implementation. RPA/LFA systems require multiple modified primers, modified antibodies, and chromogenic particles. These requirements increase the complexity of these methods, and thus may limit their clinical feasibility [28].

DNA–protein crosslinking refers to the covalent binding of proteins to DNA, leading to the formation of a complex between the two molecules [29,30]. In recent years, DNA–protein conjugates have been extensively used in biosensing, biomedical applications, and nanofabrication [31,32]. They also exhibit greater tolerance to variations in pH and ion concentration and are less susceptible to denaturation, providing significant application advantages over avidin. This system is simpler, more stable, and has lower operational costs when compared to immunological detection platforms [33].

Chemiluminescence relies exclusively on inherent luminescence generated by reactions involving catalysts, enhancers, and reaction substrates, which therefore do not require external excitation [34]. Consequently, the harnessing of inherent luminescence has led to the development of fast, sensitive, and highly automatable chemiluminescence-based detection methods with potential applicability to the field of in vitro diagnosis [35]. Furthermore, chemiluminescent images can be easily adapted for capture and analysis using smartphones for on-site detection [36], thus increasing the suitability of chemiluminescent assays for field-based applications [37].

In our study, we developed a new method called RPADPCL for detecting SARS-CoV-2. This method combines RPA with a chemiluminescence assay based on DNA–protein crosslinking. The goal is to create a rapid and easily accessible point-of-care testing solution for SARS-CoV-2 detection. The RPADPCL process involves using RPA to generate biotinylated double-stranded DNA intermediates. These intermediates then undergo crosslinking when they interact with HRP-labeled streptavidin (SA), which is a molecule with four biotin binding sites. By utilizing these techniques, we aim to provide an efficient and reliable method for detecting SARS-CoV-2.

## 2. Materials and Methods

### 2.1. Materials and Chemicals

All DNA sequences used in this study (as shown in Table S1) were synthesized by Sangon Biotech (Shanghai, China). The HRP-labeled streptavidin and ampicillin were also purchased from Sangon Biotech. Luminol and *p*-iodophenol (PIP) were purchased from Aladdin (Shanghai, China). Hydrogen peroxide (H_2_O_2_) was purchased from Shenzhen chemical reagent technology Co., Ltd. (Shenzhen, China). The TwistAmp Basic kit was purchased from TwistDx (Cambridge, UK). T7 Quick High Yield RNA Transcription Kit was purchased from Beyotime (Shanghai, China). SuperScript IV kit was purchased from Thermo Fisher Scientific (Waltham, MA, USA). TIANprep Mini Plasmid Kit was purchased from TIANGEN (Beijing, China). Dynabeads M-280 Streptavidin kit was purchased from Invitrogen (Waltham, MA, USA). Ascl restriction endonuclease kit was purchased from New England BioLabs (Ipswich, MA, USA). The SARS-CoV-2 N gene plasmid (Figure S1) was synthesized by Yunzhou Biosciences Co., Ltd. (Guangzhou, China). All chemicals used were of analytical grade or higher, without further purification.

### 2.2. Instruments

The metal heater was obtained from Tianjian Bio Tech (Beijing, China). The chemiluminescence signal was measured using a chemiluminescence analyzer (BPCL-2-TGC, Guangzhou Microphotonics Tech, Guangzhou, China). Chemiluminescence imaging was carried out using a Honor play4 pro smartphone (Huawei, Shenzhen, China). All gel images were taken using a BioRad Imaging System (Hercules, CA, USA). Ultra-pure water prepared through the Milli-Q Ultra-Pure Water System (Millipore, Burlington, MA, USA).

### 2.3. Preparation of the SARS-CoV-2 N Gene Plasmid

The cultivation of the *E. coli*-containing SARS-CoV-2 N gene plasmid was performed in a biosafety cabinet. A monoclonal colony separated on LB solid medium was selected and added to LB liquid medium with 100 mg/mL ampicillin solution. The LB liquid medium was put into a shaker at 37 °C, 180–200 rpm for 12–16 h, and then the plasmid was extracted according to the instructions of the kit.

### 2.4. Preparation of the IVT RNA

The enzyme digestion reaction was performed to linearize the plasmid using Ascl digestion. After the reaction was completed, the reaction product was purified using DNA purification kit, and then the purity and concentration of the product were determined using the NanoDrop system. After plasmid linearization, RNA of SARS-CoV-2 N gene was synthesized using the IVT kit. After incubation at 37 °C for 4 h, an appropriate amount of DNase was incubated for 30 min to remove linear DNA. Then, the RNA was purified using a phenol/chloroform method.

### 2.5. Preparation of RPA Products

An RPA reaction was performed according to the TwistAMP Basic RPA kit, which contains 29.5 μL rehydration solution, 14 mM magnesium acetate, 300 nM forward/reverse primer, and lyophilized enzymes. Then, 1 μL of target DNA solution was added and thoroughly mixed, followed by incubation at 42 °C for 30 min. When detecting RNA, the IVT RNA should be reverse-transcribed.

### 2.6. Agarose Gel Electrophoresis Analysis

The reaction products were first pre-incubated with 1x loading buffer, and then run on 1% agarose gel in 1x TAE buffer. After electrophoresis at 90 v for about 40 min, gel images were taken using the BioRad imaging system.

### 2.7. SARS-CoV-2 Detection

Firstly, different concentrations of targets (SARS-CoV-2 N gene plasmid with 1000, 500, 300, 200, 100, 50, 10, and 1 copy, respectively) were used for RPA according to the kit. After the RPA reaction for about 30 min, HRP-labeled streptavidin was added and incubated at room temperature for 15 min to form a crosslinked product. Then, an appropriate amount of streptavidin-labeled MB was added to the solution, and incubation continued for 15 min to capture and enrich the crosslinked product. Subsequently, the product was separated and washed three times with PBS to remove the excess HRP and finally re-suspended in PBS. Next, 0.5 mM luminol, 1 mM PIP, 0.1 M sodium bicarbonate, and 10 mM H_2_O_2_ were added to produce the chemiluminescence signals. A smartphone was used for chemiluminescence imaging was captured by smartphone. Additionally, chemiluminescence signal was recorded at 425 nm using a chemiluminescence analyzer. As for SARS-CoV-2 IVT RNA detection, different concentrations of targets were used for RPA according to the kit, followed by a chemiluminescence analysis as mentioned above.

### 2.8. Specificity Analysis of SARS-CoV-2 Detection

The specificity of the detection method is crucial for its practical application value. To perform a specificity analysis, different commercial plasmids were used. The concentration of SARS-CoV-2 plasmid DNA and other various interfering plasmid DNA (plasmid with actin DNA (ACTN), vaccinia virus (VACPV) F3L, Middle East respiratory syndrome coronavirus (MERS-CoV), SARS-CoV-2) was equivalent to 100 copies. Finally, the chemiluminescence analysis was performed using the same method as mentioned above.

### 2.9. SARS-CoV-2 Detection in Complex Matrix Systems Simulating Clinical Specimens

In order to further explore the application scenarios of this method, a spiked sample was created by adding 10% human saliva (diluted in reaction buffer) or VTM (Beyotime Biotech. Shanghai, China) to achieve a concentration of 0.5 pM for the SARS-CoV-2 plasmid. Subsequently, gradient dilution was carried out on the sample to reach the desired concentration using a reaction buffer. The detection method was then applied to analyze the sample and determine the recovery rate. Then, the chemiluminescence analysis was performed using the same method as mentioned above.

## 3. Results and Discussion

### 3.1. Working Principle of RPADPCL

**Figure 1:**
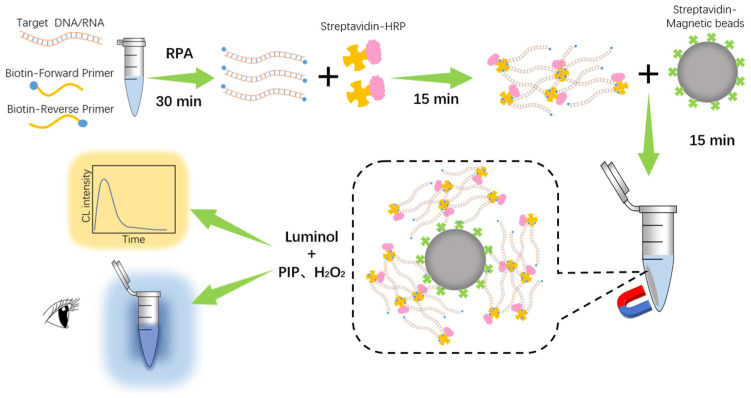
Schematic of the detection of SARS-CoV-2 based on RPA and DNA–protein crosslinking.

The working principle of the RPADPCL biosensing platform incorporating RPA reaction and target DNA–protein-crosslinking-enhanced chemiluminescence for SARS-CoV-2 detection is shown in Figure 1. Firstly, the forward primers and reverse primers modified with biotin in both 5′ ends were constructed. When a sample contains the target, the RPA process proceeds and generates a large number of amplified DNA products with biotin-labelled 5′ ends. The addition of HRP-labeled streptavidin (SA) to the system subsequently facilitated efficient binding between biotin and SA, resulting in swift crosslinking between RPA products and HRP-SA within a brief timeframe (≤15 min). In the next step, SA-labeled magnetic beads (MBs) were added to the system to capture the DNA–protein-crosslinked products. Afterwards, MBs with bound crosslinked products were magnetically separated and washed to remove unbound components. Then, the luminol substrate solution containing PIP and H_2_O_2_ was added to the re-suspended system. The mixture was catalyzed by HRP and emitted a significant chemiluminescent signal.

### 3.2. Feasibility Analysis

**Figure 2:**
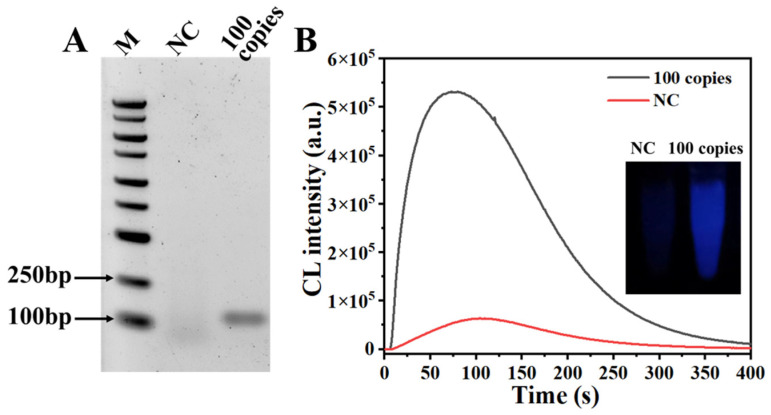
Feasibility analysis of RPADPCL. (**A**) Verification of RPA products by 1% agarose gel electrophoresis; (**B**) chemiluminescence detection and imaging.

To verify the feasibility of our RPADPCL method, a linearized plasmid containing a SARS-CoV-2 N gene sequence was used as an RPA template. As shown in Figure 2A, the lane loaded with the product generated by the RPA of the linearized plasmid template (100 copies) displayed a band with a molecular weight of about 120 bp, demonstrating effective target amplification. As previously reported, PIP has been shown to significantly increase luminescence intensity as a chemiluminescence enhancer [38,39]. In this study, we evaluated the enhanced effect of PIP on RPADPCL signal output. In Figure S2, the addition of PIP to the reaction resulted in the emission of a markedly stronger chemiluminescent signal by the positive control sample. However, in the absence of PIP, neither the positive nor the negative control produced significant chemiluminescent signals. In subsequent experiments, we introduced PIP in our detecting system. When subjected to DPCL detection, the chemiluminescent peak obtained for the sample containing the RPA DNA product possessed a signal intensity that was significantly greater than that of the negative control sample (Figure 2B). Additionally, the chemiluminescence emitted by the sample was visible to the naked eye and was clearly blue in color when viewed in a dark environment. In contrast, the negative control sample produced an extremely weak signal. These results strongly support the suitability of the RPADPCL method for SARS-CoV-2 detection.

### 3.3. Effect of Different Sample Addition Orders of SA-HRP and SA-MB on Signal Output

**Figure 3:**
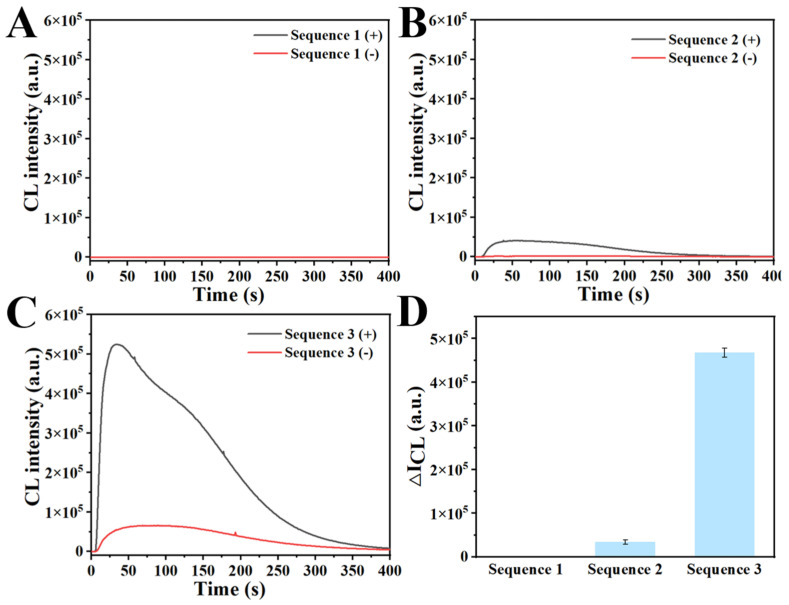
Effect of different sample addition orders of SA-HRP and SA-MB on signal output. (**A**) Order 1: SA-MB was first added to RPA products for incubation, and then SA-HRP was added for incubation. (**B**) Order 2: SA-MB and SA-HRP were mixed first and then added together into RPA products for incubation. (**C**) Order 3: SA-HRP was first added to RPA products for incubation, and then SA-MB was added for incubation. (**D**) Comparison of detection signals in different sample addition orders. All data are expressed as mean ± standard deviation (SD). Each measurement was conducted in triplicate.

To further determine the necessity of the two-step incubation of SA-HRP and SA-MB in the experimental process, three different addition orders were carried out. Figure 3A shows the result of addition order 1 (SA-MB was first added to RPA products for incubation, and then SA-HRP was added for incubation). Figure 3B shows the result of addition order 2 (SA-MB and SA-HRP were mixed first and then added together into RPA products for incubation). Figure 3C shows the result of addition order 3 (SA-HRP was first added to RPA products for incubation, and then SA-MB was added for incubation). Figure 3A,B shows no obvious signal intensity. This phenomenon may stem from two factors. Firstly, the larger volume of SA-MB compared to RPA products and SA-HRP, along with its steric hindrance, could lead to greater interference in the binding of RPA products and SA-HRP. Additionally, since SA-MB is introduced to the system first, RPA products may bind to it in advance, leading to a reduction in exposed biotin sites. Consequently, this may impede the efficient crosslinking promotion once SA-HRP is added. As shown in Figure 3C,D, a significant signal can be observed. Therefore, this method verified the necessity of separate incubation steps for SA-HRP and SA-MB. In the subsequent experiments, addition order 3 was adopted.

### 3.4. Optimization of Reaction Conditions to Enhance RPADPCL Target Detection Performance

**Figure 4:**
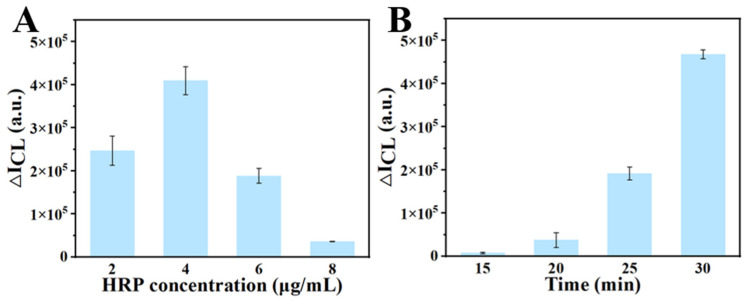
Optimization of the concentration of SA-HRP (**A**) and RPA reaction time (**B**). All data were expressed as mean ± standard deviation (SD). Each measurement was conducted in triplicate.

To enhance RPADPCL detection performance, we focused on optimizing two key parameters: SA-HRP concentration and RPA reaction time. Figure 4A shows the effect of SA-HRP concentration on signal output and demonstrated peak signal strength at the SA-HRP concentration of 4 μg/mL. However, further increases in SA-HRP concentration resulted in significant decreases in signal strength that were likely due to an excess of unbound SA-HRP in the system. Consequently, an SA-HRP concentration of 4 μg/mL was used in subsequent experiments. Figure 4B shows the effect of RPA reaction time on signal output. The results reveal that RPA reaction times of 15 or 20 min yielded weak chemiluminescent signals. However, extending the RPA reaction time from 20 to 30 min resulted in a continuous increase in signal strength, with a significant signal enhancement observed at the 30 min time point. Therefore, a 30 min RPA reaction time was selected for subsequent experiments.

### 3.5. Analytical Sensitivity of RPADPCL for the Detection of Plasmids Containing the SARS-CoV-2 N Gene Sequence

**Figure 5:**
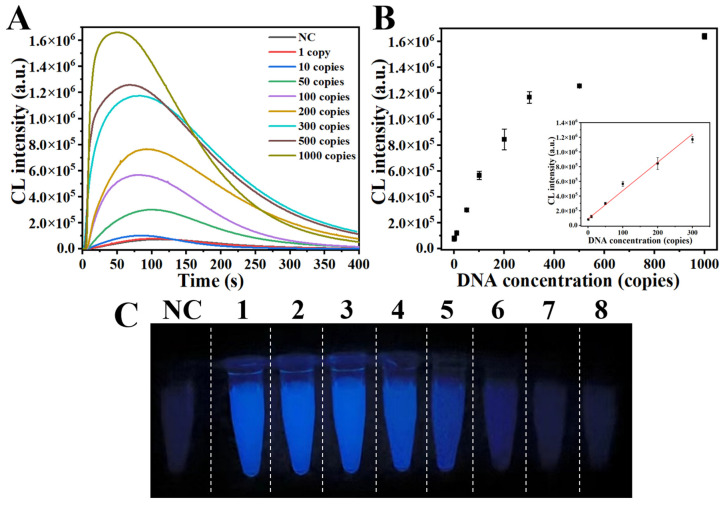
Analytical sensitivity of RPADPCL for SARS-CoV-2 N gene plasmid detection. (**A**) Chemiluminescence spectra of different concentrations of SARS-CoV-2 N gene plasmid. (**B**) The linear relationship between different concentrations of SARS-CoV-2 N gene plasmid and chemiluminescence intensity. (**C**) Visualization imaging of different concentrations of SARS-CoV-2 N gene plasmid. NC: Negative control. 1–8: SARS-CoV-2 N gene plasmid with 1000, 500, 300, 200, 100, 50, 10, and 1 copies, respectively. All data are expressed as mean ± SD. Each measurement was conducted in triplicate.

Due to the presence of only trace amounts of SARS-CoV-2 in clinical samples, we assessed RPADPCL sensitivity under optimal conditions using various concentrations of a plasmid containing a SARS-CoV-2 N gene sequence (Figure S3). Through an analysis of chemiluminescence spectra, it was observed that the signal strength progressively increased as the concentration of the plasmid in the system was raised from 1 copy to 1000 copies (Figure 5A and Figure S4). As a result of this observation, we proceeded to establish a standard curve by plotting the plasmid concentration and chemiluminescence intensity at 425 nm (Figure 5B). The resulting standard curve revealed a linear relationship between these variables when the plasmid copy number fell within the range of 1 to 300 copies. The equation describing the line of best fit for the linear portion of the standard curve was determined to be y = 3850.3x + 89,446.6 and found to possess a high *R^2^* value of 0.984. According to the 3σ/k rule, the limit of detection (LOD) was determined to be 6 copies. As depicted in Figure 5C, it can be clearly seen that when the plasmid copy number approached 50 copies, a significant increase in luminescence intensity was visualized as compared to that of the negative control. In summary, these results suggest that the proposed RPADPCL assay has great potential for achieving the sensitive detection of SARS-CoV-2 in clinical samples.

As compared with other existing methods (Table S2), the proposed method offers the advantages of speed, sensitivity, and specificity, as well as intuitive and easy implementation, requiring no external excitation input for chemiluminescent signal generation. Collectively, these results demonstrate that the RPADPCL assay may be suitable for detecting SARS-CoV-2 in clinical samples.

### 3.6. Analytical Sensitivity of RPADPCL for SARS-CoV-2 IVT RNA Detection

**Figure 6:**
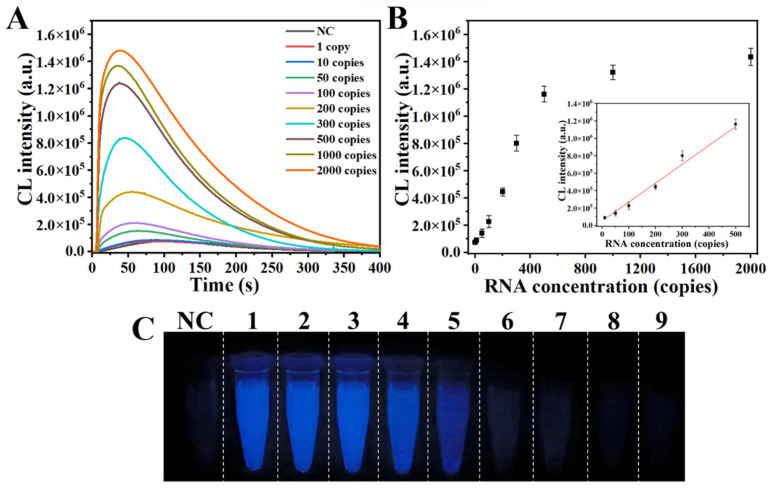
Analytical sensitivity of RPADPCL for SARS-CoV-2 IVT RNA detection. (**A**) Chemiluminescence spectra of different concentrations of SARS-CoV-2 IVT RNA. (**B**) The linear relationship between different concentrations of SARS-CoV-2 IVT RNA and chemiluminescence intensity. (**C**) Visualization imaging of different concentrations of SARS-CoV-2 IVT RNA. NC: Negative control. 1–9: SARS-CoV-2 IVT RNA with 2000, 1000, 500, 300, 200, 100, 50, 10, and 1 copies, respectively. All data are expressed as mean ± SD. Each measurement was conducted in triplicate.

To assess RPADPCL sensitivity in detecting SARS-CoV-2, RPADPCL assays were conducted using various concentrations of SARS-CoV-2 IVT RNA (Figure S5). An analysis of chemiluminescence intensities revealed increasing signal strengths as the concentration of IVT RNA increased from 1 to 2000 copies (Figure 6A and Figure S6). A standard curve between IVT RNA concentration and chemiluminescence intensity revealed a linear relationship when the SARS-CoV-2 IVT RNA copy number ranged from 1 to 500 copies (Figure 6B). The line of best fit for the linear region of the standard curve is described in the equation y = 2162.5x + 5480.95 and possesses a high *R^2^* value of 0.985. According to the 3σ/k rule, the LOD was calculated to be 15 copies. Meanwhile, Figure 6C illustrates that there was a significant increase in luminescence intensity when the concentration of IVT RNA reached 200 copies. Therefore, these findings show the potential of our method for rapid and efficient virus detection.

### 3.7. Specificity Analysis of RPADPCL

**Figure 7:**
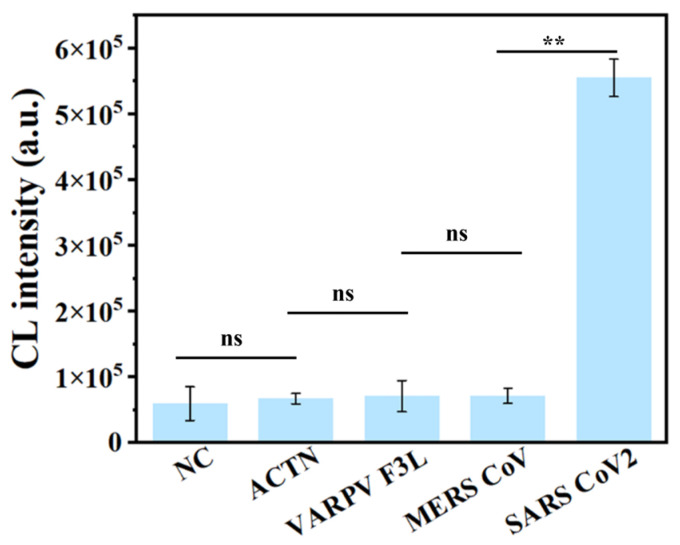
The specificity analysis of RPADPCL. The concentrations of SARS-CoV-2 plasmid DNA and other various interfering viral plasmid DNA (ACTN, VARPV F3L, MERS CoV, SARS-CoV-2) were 100 copies. NC: Negative control. All data are expressed as mean ± SD. Each measurement was conducted in triplicate. ns: *p* > 0.05, ** *p* < 0.01.

Specificity is another key feature of RPADPCL for SARS-CoV-2 detection. The practical application of the RPADPCL method relies on its specificity, which generally depends on the homology between the target template and primer. As shown in Figure 7, we assessed the detection specificity of the method by detecting different types of nucleic acid targets using an equal number of target template-containing plasmid molecules per reaction (100 copies). Notably, in the presence of the plasmid containing the gene encoding the SARS-CoV-2 N protein, more intense detection signals were obtained compared to those of other targets. This result indicates that the RPADPCL assay exhibits good specificity in distinguishing the SARS-CoV-2 N protein gene from other targets.

### 3.8. Detection of SARS-CoV-2 in Simulated Clinical Samples

In this study, we explored the adaptability of our RPADPCL assay to the detection of targets present in complex matrix systems simulating clinical specimens, such as 10% human saliva or VTM spiked with either a plasmid containing a SARS-CoV-2 N gene sequence or SARS-CoV-2 IVT RNA. As shown in Table 1, target recovery rates in 10% human saliva ranged from 95% to 103%. Similarly, target recovery rates ranging from 86.4% to 96% were obtained in VTM, which is a viral storage solution used to stabilize SARS-CoV-2 virus in patient throat and nose swabs during transport to the testing lab, as shown in Table 2. These results affirm that the RPADPCL method consistently delivered good recovery rates when used to detect targets within complex matrix systems. Thus, this method holds great potential for use in detecting targets in clinical samples.

## 4. Conclusions

Here, a simple visual and point-of-care strategy for SARS-CoV-2 detection using RPADPCL was developed. RPA was utilized to generate biotinylated double-stranded products as crosslinking intermediates for the crosslinking of HRP-labeled streptavidin. After adding a chemiluminescent substrate, chemiluminescence detection was performed. Under optimal conditions, the LOD of SARS-CoV-2 N gene plasmid and IVT RNA were determined to be 6 copies and 15 copies, respectively. The linear ranges were 1–300 copies and 10–500 copies, respectively. The specificity analysis of RPADPCL was able to effectively distinguish different viral plasmid DNA, thus possessing good specificity. Meanwhile, this method can also be integrated with smartphones to capture and analyze chemiluminescence signals. In clinical applications, 10% human saliva and VTM media were used for the preparation of spiked samples, and good recovery rates were obtained, respectively, verifying the effectiveness of this method in detecting SARS-CoV-2 in complex matrix systems. Thus, this novel RPADPCL has the potential for simple, visual, on-site SARS-CoV-2 detection, helping to prevent its transmission.

While the RPADPCL method has shown promise in detecting targets in simulated clinical samples, there are still several limitations to this study. One key limitation is the need for further validation of the method using actual clinical samples to fully assess its detection performance in a clinical setting. Additionally, it is necessary to investigate whether this method can be effectively used for the on-site detection of other complex matrices such as whole blood and plasma. Another limitation is that the current two-step incubation and separation process may not be the most efficient or streamlined approach. Future research should focus on designing a one-pot method that simplifies this process and improves efficiency. Overall, while the RPADPCL method shows great potential, further research is needed to address these limitations and fully evaluate its utility in clinical settings.

## Figures and Tables

**Table 1:** Recovery experiment for SARS-CoV-2 N gene plasmid in 10% human saliva.

Sample No.	Spiked (Copies)	CL Intensity (a.u.)	Found ^a^ (Copies)	Recovery ^b^ (%)	RSD ^c^ (%)
1	100	4.87 × 10^5^	95 ± 15	95%	15.8%
		4.93 × 10^5^			
		3.87 × 10^5^			
2	300	1.29 × 10^6^	309 ± 35	103%	11.3%
		1.41 × 10^6^			
		1.14 × 10^6^			

Notes: ^a^ Found are expressed as Mean ± SD (n = 3). ^b^ Recovery (%) = Found/Spiked × 100%. ^c^ relative standard deviation, RSD (%) = SD/Mean × 100%.

**Table 2:** Recovery experiment for ITV RNA in VTM.

Sample No.	Spiked (Copies)	CL Intensity (a.u.)	Found ^a^ (Copies)	Recovery ^b^ (%)	RSD ^c^ (%)
1	100	2.67 × 10^5^	96 ± 13	96%	13.5%
		2.92 × 10^5^			
		2.32 × 10^5^			
2	500	9.51 × 10^5^	432 ± 21	86.4%	4.9%
		1.04 × 10^6^			
		9.79 × 10^5^			

Notes: ^a^ Found are expressed as Mean ± SD (n = 3). ^b^ Recovery (%) = Found/Spiked × 100%. ^c^ relative standard deviation, RSD (%) = SD/Mean × 100%.

## Data Availability

The data presented in this study are available in the article and Supplementary Materials.

## Supplementary Materials

The following supporting information can be downloaded at: https://www.mdpi.com/article/10.3390/bios14030135/s1, Table S1. Detailed information of the oligonucleotide sequences used in this study; Figure S1. The designed SARS-CoV-2 N gene plasmid map; Figure S2. Effect of the PIP enhancer on RPADPCL chemiluminescent signal strength. Figure S3. Identification of SARS-CoV-2 N gene plasmid by 1% agarose gel electrophoresis; Figure S4. Identification of RPA products for SARS-CoV-2 N gene plasmid detection by 1% agarose gel electrophoresis; Figure S5. Identification of SARS-CoV-2 IVT RNA by 1% agarose gel electrophoresis; Figure S6. Identification of RPA products for IVT RNA detection by 1% agarose gel electrophoresis; Table S2. Comparison between the proposed method and other RPA-based SARS-CoV-2 detection methods [27,40,41,42,43,44,45,46,47,48,49,50].
