# Supplementary material for: Simple, Visual, Point-of-Care SARS-CoV-2 Detection Incorporating Recombinase Polymerase Amplification and Target DNA–Protein Crosslinking Enhanced Chemiluminescence
Source: Biosensors (Basel). 2024 Mar 6;14(3):135. doi: 10.3390/bios14030135 (PMC10968138; doi:10.3390/bios14030135)
Supplement: Supplementary file 1 [file biosensors-14-00135-s001.zip › biosensors-2702183-supplementary.pdf]

Simple, Visual, Point-of-Care SARS-CoV-2 Detection Incorporating Recombinase Polymerase Amplification and Target DNA–Protein Crosslinking Enhanced Chemiluminescence

CONTENTS

Table S1. Detailed information of the oligonucleotide sequences used in this study. S2

Figure S1. The designed SARS-CoV-2 N gene plasmid map. S3

Figure S2. Effect of the PIP enhancer on RPADPCL chemiluminescent signal strength S4

Figure S3. Identification of SARS-CoV-2 N gene plasmid by 1% agarose gel electrophoresis. S5

Figure S4. Identification of RPA products for SARS-CoV-2 N gene plasmid detection by 1% agarose gel electrophoresis. S5

Figure S5. Identification of SARS-CoV-2 IVT RNA by 1% agarose gel electrophoresis. S5

Figure S6. Identification of RPA products for IVT RNA detection by 1% agarose gel electrophoresis. S6

Table S2. Comparison between the proposed method and other RPA-based SARS-CoV-2 detection methods. S7

Reference S8

Table S1. Detailed information of the oligonucleotide sequences used in this study.

Names	Sequences (5'-3')
SARS-CoV-2 N gene	ATGTCTGATAATGGACCCCAAAATCAGCGAAATGCACCCCGCATTACGTTTGGTGGA<br>CCCTCAGATTCAACTGGCAGTAACCAGAATGGAGAACGCAGTGGGGCGCGATCAAA<br>ACAACGTCGGCCCCAAGGTTTACCCAATAATACTGCGTCTTGGTTCACCGCTCTCAC<br>TCAACATGGCAAGGAAGACCTTAAATTCCCTCGAGGACAAGGCGTTCCAATTAACA<br>CCAATAGCAGTCCAGATGACCAAATTGGCTACTACCGAAGAGCTACCAGACGAATT<br>CGTGGTGGTGACGGTAAAATGAAAGATCTCAGTCCAAGATGGTATTTCTACTACCTA<br>GGAACTGGGCCAGAAGCTGGACTTCCCTATGGTGCTAACAAAGACGGCATCATATG<br>GGTTGCAACTGAGGGAGCCTTGAATACACCAAAAGATCACATTGGCACCCGCAATC<br>CTGCTAACAATGCTGCAATCGTGCTACAACTTCCTCAAGGAACAACATTGCCAAAAG<br>GCTTCTACGCAGAAGGGAGCAGAGGCGGCAGTCAAGCCTCTTCTCGTTCCTCATCAC<br>GTAGTCGCAACAGTTCAAGAAATTCAACTCCAGGCAGCAGTAGGGGAACTTCTCCT<br>GCTAGAATGGCTGGCAATGGCGGTGATGCTGCTCTTGCTTTGCTGCTGCTTGACAGA<br>TTGAACCAGCTTGAGAGCAAAATGTCTGGTAAAGGCCAACAACAACAAGGCCAAAC<br>TGTCACTAAGAAATCTGCTGCTGAGGCTTCTAAGAAGCCTCGGCAAAAACGTACTGC<br>CACTAAAGCATACAATGTAACACAAGCTTTCGGCAGACGTGGTCCAGAACAAACCC<br>AAGGAAATTTTGGGGACCAGGAACTAATCAGACAAGGAACTGATTACAAACATTGG<br>CCGCAAATTGCACAATTTGCCCCCAGCGCTTCAGCGTTCTTCGGAATGTCGCGCATT<br>GGCATGGAAGTCACACCTTCGGGAACGTGGTTGACCTACACAGGTGCCATCAAATT<br>GGATGACAAAGATCCAAATTTCAAAGATCAAGTCATTTTGCTGAATAAGCATATTGA<br>CGCATACAAAACATTCCCACCAACAGAGCCTAAAAAGGACAAAAAGAAGAAGGCT<br>GATGAAACTCAAGCCTTACCGCAGAGACAGAAGAAACAGCAAACTGTGACTCTTCT<br>TCCTGCTGCAGATTTGGATGATTTCTCCAAACAATTGCAACAATCCATGAGCAGTGC<br>TGACTCAACTCAGGCCTAA
SARS-2 FP	CAACTTCCTCAAGGAACAACATTGCCAAAA
SARS-2 RP	TGGAGTTGAATTTCTTGAACTGTTGCGACT
SARS-2 FP-biotin	biotin-CAACTTCCTCAAGGAACAACATTGCCAAAA
SARS-2 RP-biotin	biotin-TGGAGTTGAATTTCTTGAACTGTTGCGACT

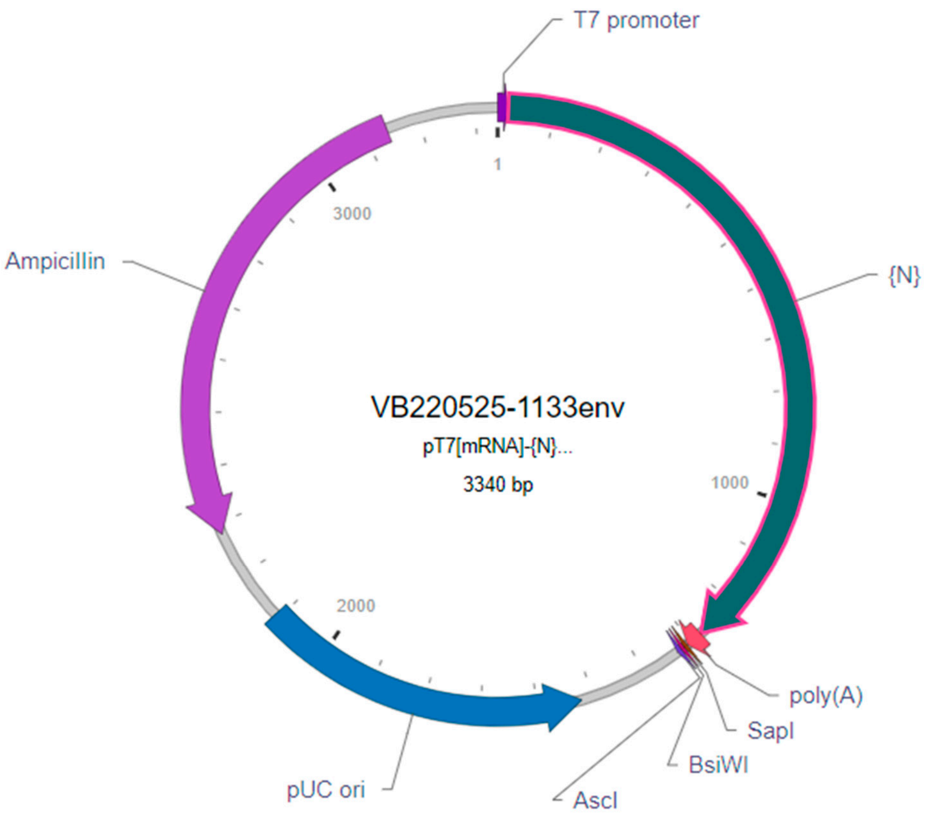

Figure S1. The designed SARS-CoV-2 N gene plasmid map.

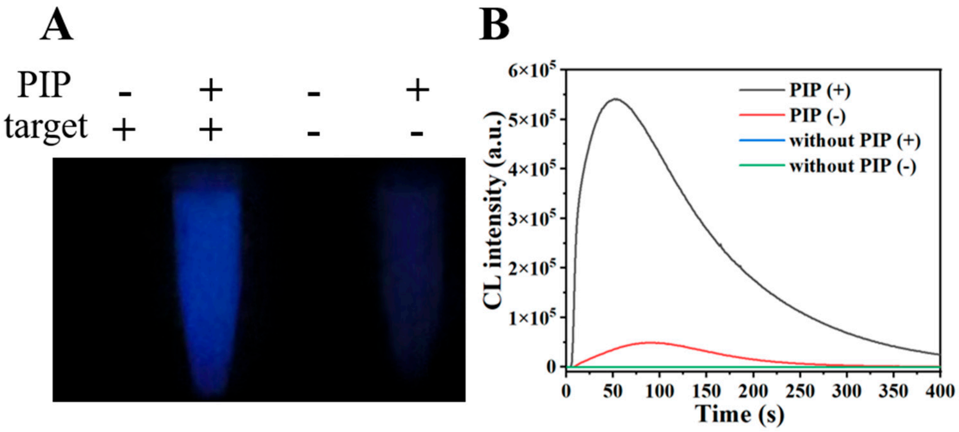

Figure S2. Effect of the PIP enhancer on RPADPCL chemiluminescent signal strength.

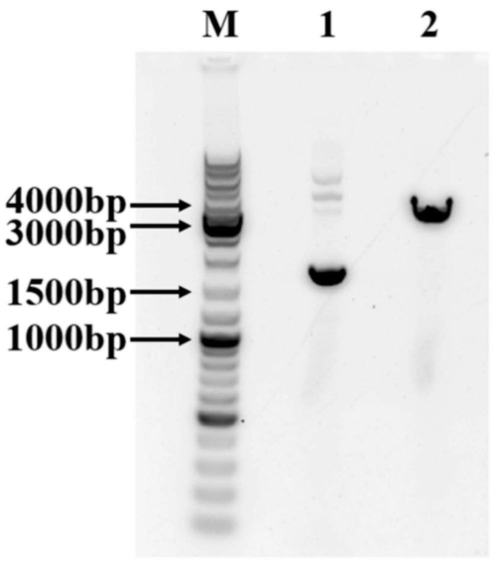

Figure S3. Identification of SARS-CoV-2 N gene plasmid by 1% agarose gel electrophoresis. M：DNA marker; Lane 1：The designed SARS-CoV-2 N gene plasmid. Lane 2：The plasmid after AscI enzyme digestion.

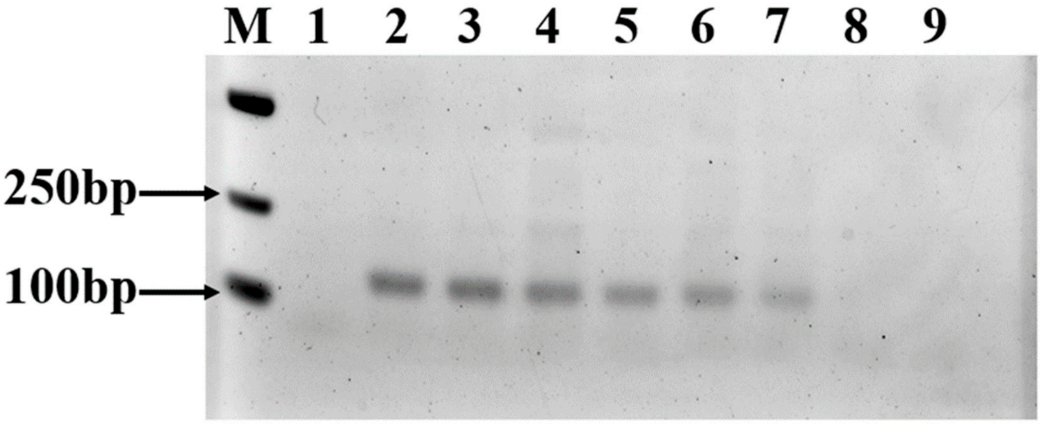

Figure S4. Identification of RPA products for SARS-CoV-2 N gene plasmid detection by 1% agarose gel electrophoresis. M：DNA marker; Lane 1: negative control; Lane 2-9: The concentration of SARS-CoV-2 N gene plasmid was 1000, 500, 300, 200, 100, 50, 10, 1 copies, respectively.

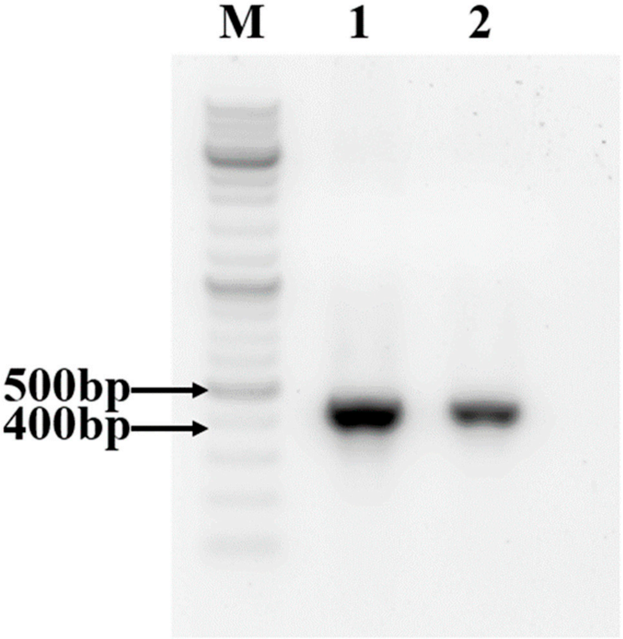

Figure S5. Identification of SARS-CoV-2 IVT RNA by 1% agarose gel electrophoresis. M：DNA marker; Lane 1： The IVT RNA synthetized by RiboMAX Large Scale RNA Production System Kit. Lane 2： The IVT RNA synthetized byT7 Quick High Yield RNA Transcription Kit.

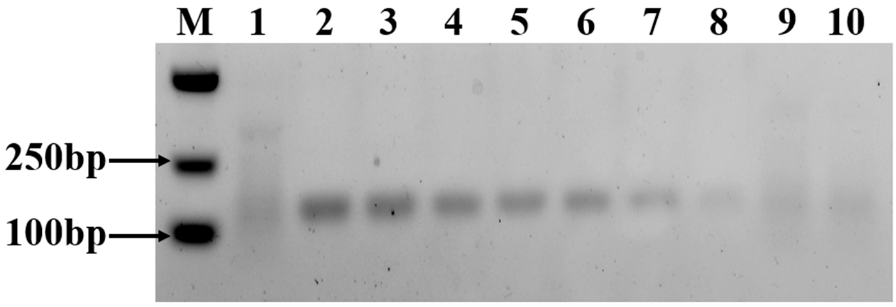

Figure S6. Identification of RPA products for IVT RNA detection by 1% agarose gel electrophoresis. M：DNA marker; Lane 1: negative control; Lane 2-10: The concentration of SARS-CoV-2 N gene plasmid was 2000, 1000, 500, 300, 200, 100, 50, 10, 1 copies, respectively.

Table S2. Comparison between the proposed method and other RPA-based SARS-CoV-2 detection methods.

No.	Methods	Target genes	Sensitivity (LOD)	Quantification	Visualization	Detection time	Ref.
1	Strip	N/ORF1ab gene	10 copies	×	√	60 min	[1]
2	CRISPR/Cas9-mediated strip	E/ORF1ab gene	100 copies	×	√	58 min	[2]
3	Strip	N/S gene	10 copies	×	√	45 min	[3]
4	Microfluidic-integrated strip	N gene	30 copies	×	√	20 min	[4]
5	Strip	N gene	35.4 copies	×	√	45 min	[5]
6	CRISPR/Cas fluorometry	N/ORF1ab gene	2 copies	√	×	50 min	[6]
7	Colorimetric CRISPR/Cas12a assay	N/ORF1ab gene	1 copy	√	×	240 min	[7]
8	Real-time RPA	N gene	10 copies	√	×	27 min	[8]
9	Real-time RPA	ORF1ab/S gene	10 copies	√	×	24 min	[9]
10	Real-time RPA	N/E/RdRP gene	15 copies	√	×	15 min	[10]
11	Fluorescent strip	E/RdRP gene	9.5 copies	√	×	30 min	[11]
12	CRISPR/Cas-based lab-on-paper	N/S gene	100 copies	√	×	60 min	[12]
13	Chemiluminometry	N gene	15 copies	√	√	60 min	This work

Reference

1. Sun, Y.; Qin, P.; He, J.; Li, W.; Shi, Y.; Xu, J.; Wu, Q.; Chen, Q.; Li, W.; Wang, X., Rapid and simultaneous visual screening of SARS-CoV-2 and influenza virufses with customized isothermal amplification integrated lateral flow strip. *Biosens. Bioelectron.* **2022,** 197, 113771.
2. Xiong, E.; Jiang, L.; Tian, T.; Hu, M.; Yue, H.; Huang, M.; Lin, W.; Jiang, Y.; Zhu, D.; Zhou, X., Simultaneous dual-gene diagnosis of SARS-CoV-2 based on CRISPR/Cas9-mediated lateral flow assay. *Angew. Chem. Int. Edit.* **2021,** 60, (10), 5307-5315.
3. Qian, J.; Boswell, S. A.; Chidley, C.; Lu, Z.-x.; Pettit, M. E.; Gaudio, B. L.; Fajnzylber, J. M.; Ingram, R. T.; Ward, R. H.; Li, J. Z., An enhanced isothermal amplification assay for viral detection. *Nat. Commun.* **2020,** 11, (1), 5920.
4. Liu, D.; Shen, H.; Zhang, Y.; Shen, D.; Zhu, M.; Song, Y.; Zhu, Z.; Yang, C., A microfluidic-integrated lateral flow recombinase polymerase amplification (MI-IF-RPA) assay for rapid COVID-19 detection. *Lab Chip* **2021,** 21, (10), 2019-2026.
5. Shelite, T. R.; Uscanga-Palomeque, A. C.; Castellanos-Gonzalez, A.; Melby, P. C.; Travi, B. L., Isothermal recombinase polymerase amplification-lateral flow detection of SARS-CoV-2, the etiological agent of COVID-19. *J. Virol. Methods* **2021,** 296, 114227.
6. Huang, Z.; Tian, D.; Liu, Y.; Lin, Z.; Lyon, C. J.; Lai, W.; Fusco, D.; Drouin, A.; Yin, X.; Hu, T., Ultra-sensitive and high-throughput CRISPR-p owered COVID-19 diagnosis. *Biosens. Bioelectron.* **2020,** 164, 112316.
7. Zhang, W. S.; Pan, J.; Li, F.; Zhu, M.; Xu, M.; Zhu, H.; Yu, Y.; Su, G., Reverse transcription recombinase polymerase amplification coupled with CRISPR-Cas12a for facile and highly sensitive colorimetric SARS-CoV-2 detection. *Anal. Chem.* **2021,** 93, (8), 4126-4133.
8. Wu, T.; Ge, Y.; Zhao, K.; Zhu, X.; Chen, Y.; Wu, B.; Zhu, F.; Zhu, B.; Cui, L., A reverse-transcription recombinase-aided amplification assay for the rapid detection of N gene of severe acute respiratory syndrome coronavirus 2 (SARS-CoV-2). *Virology* **2020,** 549, 1-4.
9. Xue, G.; Li, S.; Zhang, W.; Du, B.; Cui, J.; Yan, C.; Huang, L.; Chen, L.; Zhao, L.; Sun, Y., Reverse-transcription recombinase-aided amplification assay for rapid detection of the 2019 novel coronavirus (SARS-CoV-2). *Anal. Chem.* **2020,** 92, (14), 9699-9705.
10. El Wahed, A. A.; Patel, P.; Maier, M.; Pietsch, C.; Rüster, D.; Böhlken-Fascher, S.; Kissenkötter,

J.; Behrmann, O.; Frimpong, M.; Diagne, M. M., Suitcase lab for rapid detection of SARS-CoV-2 based on recombinase polymerase amplification assay. *Anal. Chem.* **2021,** 93, (4), 2627-2634.

11. Cherkaoui, D.; Huang, D.; Miller, B. S.; Turbé, V.; McKendry, R. A., Harnessing recombinase polymerase amplification for rapid multi-gene detection of SARS-CoV-2 in resource-limited settings. *Biosens. Bioelectron.* **2021,** 189, 113328.

12. Yin, K.; Ding, X.; Li, Z.; Sfeir, M. M.; Ballesteros, E.; Liu, C., Autonomous lab-on-paper for multiplexed, CRISPR-based diagnostics of SARS-CoV-2. *Lab Chip* **2021,** 21, (14), 2730-2737.
